# Identification of the Interface in a Binary Complex Plasma Using Machine Learning

**DOI:** 10.3390/jimaging5030036

**Published:** 2019-03-12

**Authors:** He Huang, Mierk Schwabe, Cheng-Ran Du

**Affiliations:** 1 College of Science, Donghua University, Shanghai 201620, China; 2 Institut für Materialphysik im Weltraum, Deutsches Zentrum für Luft- und Raumfahrt (DLR), 82234 Weßling, Germany; 3 Magnetic Confinement Fusion Research Centre, Ministry of Education, Shanghai 201620, China

**Keywords:** complex plasma, machine learning

## Abstract

A binary complex plasma consists of two different types of dust particles in an ionized gas. Due to the spinodal decomposition and force imbalance, particles of different masses and diameters are typically phase separated, resulting in an interface. Both external excitation and internal instability may cause the interface to move with time. Support vector machine (SVM) is a supervised machine learning method that can be very effective for multi-class classification. We applied an SVM classification method based on image brightness to locate the interface in a binary complex plasma. Taking the scaled mean and variance as features, three areas, namely small particles, big particles and plasma without dust particles, were distinguished, leading to the identification of the interface between small and big particles.

A complex plasma is a weakly ionized gas containing small solid particles [1,2]. The particles are highly charged by collecting ions and electrons, and interact strongly with each other. This system allows experimental studies of various physical processes occurring in liquids and solids at the kinetic level [3], such as plasma crystals [4,5], acoustic waves [6], and turbulence [7,8]. A complex plasma consisting of two differently sized microparticle types is known as binary complex plasma. Under certain conditions, two types of particles can be mixed and form a glassy system [9]. Other phenomena, such as phase separation [10,11] and lane formation [12], can also be studied in such systems. Under microgravity conditions, phase separation can occur due to the imbalance of forces despite the criterion of spinodal decomposition not being fulfilled [13]. The phase separated system then allows carrying out dedicated experiments such as wave transmission [14], and interaction of spheres with differently sized particles [15].

In complex plasmas, the particle radius usually ranges from a few to hundreds of microns. Then, the particles can be illuminated by a laser and directly recorded by a video camera [16]. The recorded image sequences can be further analyzed by using tracking algorithms [17,18] and the trajectories of individual particles can be obtained. This provides a basis to study the dynamics and interactions in a multi-particle system. However, under certain conditions, a large region of interest needs to be recorded with a high recording rate. As a result, the spatial resolution has to be sacrificed with currently affordable recording technology. Therefore, advanced image recognition techniques are desirable in the research of complex plasmas.

In recent years, machine learning has been widely applied to image recognition [19,20,21], such as face recognition [22], and handwriting recognition [23,24]. Machine learning methods include many different algorithms [25], such as decision trees [26], neural networks [27], Bayesian networks [28], k-Nearest Neighbor (kNN) [29] and support vector machine (SVM) [30]. Among these algorithms, the SVM method is one of the common supervised learning models for classification and regression problems [30]. Given a training set, the aim of SVM is to find the “maximum-margin hyperplane” that divides the group of points and maximizes the distance between the hyperplane and the nearest point from either group [30]. Once the hyperplane has been established by SVM training algorithm, we can classify samples not in the training set. The SVM method has been applied in solving various practical problems, such as text and hypertext categorization [31], classification of images [32] and other scientific research projects [33,34].

We applied the SVM method to achieve automatic identification of the interface in a binary complex plasma based on the brightness of the recorded images. The experiments were performed in the PK-3 Plus Laboratory on board the International Space Station (ISS). Technical details of the setup can be found in Reference [35]. An argon plasma was produced by a capacitively coupled radio-frequency (rf) generator in push–pull mode at 13.56 MHz. We prepared a binary complex plasma by injecting two types of particles. The first type was melamine formaldehyde (MF) particles of a diameter of 2.55 μm with a mass $m_{b}=1.34\times 10^{-14}$ kg, while the second type was SiO${}_{2}$ particles of a diameter of 1.55 μm with a mass $m_{s}=3.6\times 10^{-15}$ kg. Using the quadrant view (QV) camera [35], a cross-section of the left half of the particle cloud (illuminated by a laser sheet) was recorded with a frame rate of 50 frames-per-second (fps).

Due to the gradient of plasma potential close to the chamber wall, both particle types were confined in the bulk plasma region, forming a three-dimensional (3D) cloud with a cylindrical symmetry. Figure 1a shows the left half of the cross section of the particle cloud. The cloud of small particles, big particles, and microparticle-free plasma can be easily distinguished with the naked eye. The two particle types were phase-separated due to the following reasons: First, the disparity of particle size ($\Delta d/\bar{d}\approx 0.5$) was much larger than the critical value of spinodal decomposition [10]. Second, both particle types were subjected to two forces under microgravity conditions, namely the ion drag force (directed outwards from the center of the plasma chamber) and the electric-field force (directed inwards to the center of the plasma chamber). The total force acting on the two particle types had a subtle difference depending on the particle diameter [13]. The synergistic effects of spinodal decomposition and force difference led to the instantaneous phase separation. Particularly the second effect drove the small particles into the inner part of the particle cloud and left big particles outside [14].

To identify the interface of the particle cloud, we applied the SVM method to distinguish two particle types and the background microparticle-free plasma, which defined the three possible classes. First, we prepared the training sets and defined the features. Three areas (representing three classes) were selected in one frame of the experimental video (marked by rectangles in Figure 1a). Class 1 is the small particles area, class 2 is the big particles area, and class 3 is the background plasma without dust particles. The areas are far from the interface to avoid ambiguity, and their class can be easily identified with the naked eye. To define features, we randomly selected 4 × 4 pixel tiles from the selected area. Figure 1b–d shows a part of the tile collections. Here, each tile was one sample. The mean and the variance of each sample were selected as features. As the variance was much bigger than the mean, we rescaled both features so that their magnitudes were comparable:

(1) $$\;S_{i}^{j}=\frac{x_{i}^{j}-x_{min}^{j}}{x_{max}^{j}-x_{min}^{j}}\;$$

where *i* stands for sample *i*, j=1 represents variance and j=2 represents mean. $x_{min}^{j}$ and $x_{max}^{j}$ are the minimum and maximum of the *j* feature of all samples of all classes. We labeled each sample as 1, 2, or 3 based on the area it belonged to and repeated the process for a few frames. All samples were randomly divided into two sets, namely the training set and the test set. Here, we selected 216 samples in each class for training. Table 1 shows the values of the features and labels of a few samples.

Next, we applied one of the support vector machine methods, namely the support vector classification (SVC), to the training set. The algorithm was provided by scikit-learn API [36,37]. The SVC method implemented the “one-against-one” approach for multi-class classification. The “one-against-one” approach involves constructing a machine for each pair of classes. Thus, three classifiers were constructed, each constructed by two different classes of training points, i.e., “big” or “small particles”, “big” or “particle-free”, and “small” or “particle-free”. When applied to a sample, each classifier gave one vote to the winning class, and the sample was labeled with the class having most votes [38]. The parameter C in the scikit-learn API was set to 5, which is the penalty parameter of the error term. A larger values of C allows for fewer incorrect classifications. The linear kernel type was used in the algorithm and the remaining parameters were the default parameters. More detailed instructions can be seen in scikit-learn API [36,37].

The results of the SVC classification are shown in Figure 2. Dark blue dots represent samples in the background microparticle-free plasma, light blue dots represent samples in the cloud of big particles, and dark red dots represent samples in the cloud of small particles. The classification lines are indicated by the blue and red dotted lines. When the sample is located below the blue line in Figure 2, the corresponding pixels represent the background microparticle-free plasma. When the sample is above the blue line and above the red line, the corresponding pixels belong to the cloud of big particles. When the sample is below the red line, the corresponding pixels belong to the cloud of small particles.

To evaluate the accuracy of the classification, we selected 1000 samples (with knowledge of their class) from the test set, which was set aside before the training. The accuracy was defined as the percentage of correctly classified samples out of all the selected test samples. Here, we studied the dependence of the accuracy of the SVM model on the size of the training set and the spatial resolution. As we can see in Figure 3a, with a small set of training samples, the accuracy depended on which samples we selected. On the one hand, if the randomly selected training samples represented the overall properties of the class, the resulting accuracy was high. On the other hand, if the selected samples represented only a part of the properties, the accuracy was low. This randomness led to a relatively low accuracy with big standard deviations. However, the accuracy improved quickly as the number of training samples increased. When the number of training samples exceeded 40, the accuracy approached 100%. For the classification, the spatial resolution depended on the size of each tile. Bigger tiles include more information in each tile but lead to lower spatial resolution. In Figure 3b, we show the dependence of the accuracy of the classification on the side length of each tile. With a considerable size of the training set, the accuracy already exceeded 90% when each tile included only four (2×2) pixels. This shows that the mean value of the pixel brightness alone played a significant role in classification. The accuracy rose further with the tile size. It exceeded 98% when there were more than sixteen (4×4) pixels in each tile. Comparing the results using features with (blue lines) and without (orange lines) scaling in Figure 3, we found that the scaling allowed for a reduction of the size of the training set and increased the spatial resolution of the classification.

Finally, we applied the trained algorithm to distinguish the two particle types and microparticle-free plasma in another frame of the recorded video. The original image and the results after classification are shown in Figure 4a,b, respectively. As we compare these two panels, we see that the small particles (white area), big particles (gray area), and the background microparticle-free plasma (black area) are clearly distinguished. An interface can be drawn between the small and big particles (highlighted by the red line). Here, the red line is obtained by calculating each of the demarcation points of the horizontal pixels, while the green dotted line is obtained by connecting the innermost big particle cloud pixels. The discrepancy may be caused by the presence of a third type of particles with intermediate size in the experiment run.

In summary, we applied the SVM method to achieve automatic identification of the interface in a binary complex plasma. The experiments were performed in a binary complex plasma under microgravity conditions on board the ISS, where the particle size cannot be directly deduced in the recorded images by the QV camera. The results show that this method can effectively distinguish small and big particles and the background microparticle-free plasma using the scaled mean and variance of the pixel brightness with low demand for the training samples.

## Figures and Tables

**Figure 1:**
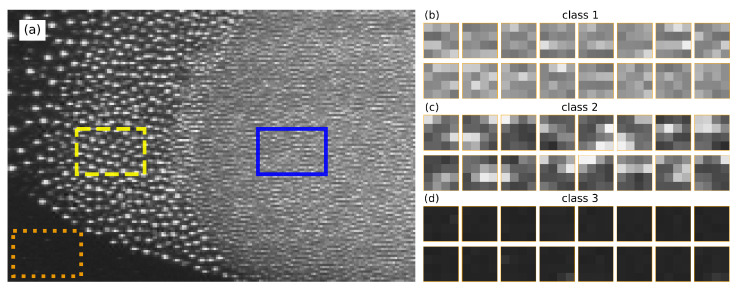
(**a**) Single image extracted from experiment recordings and the selected areas for training (highlighted by the rectangles). (**b**) Examples of samples of the small particle cloud corresponding to class 1 (highlighted by the blue solid rectangle). (**c**) Examples of samples of the big particle cloud corresponding to class 2 (highlighted by the yellow dashed rectangle). (**d**) Examples of samples of the background plasma without dust particles corresponding to class 3 (highlighted by the orange dotted rectangle). We randomly selected four by four pixel tiles from each area and calculated the mean and variance of the pixel brightness in each grid area as features for the SVM method. The grid size defines the spatial resolution.

**Figure 2:**
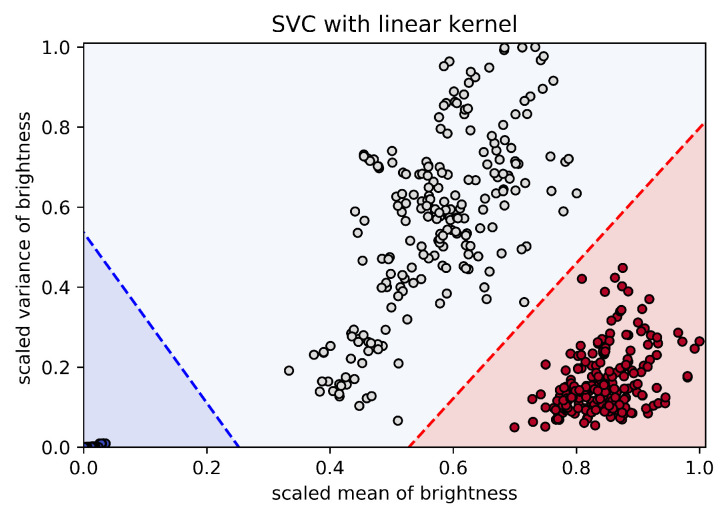
The results of the SVC classification. Dark blue dots represent samples in the background microparticle-free plasma, light blue dots represent samples of the big particle cloud, and dark red dots represent samples of the small particle cloud. The classification lines are indicated by the blue and red dotted lines.

**Figure 3:**
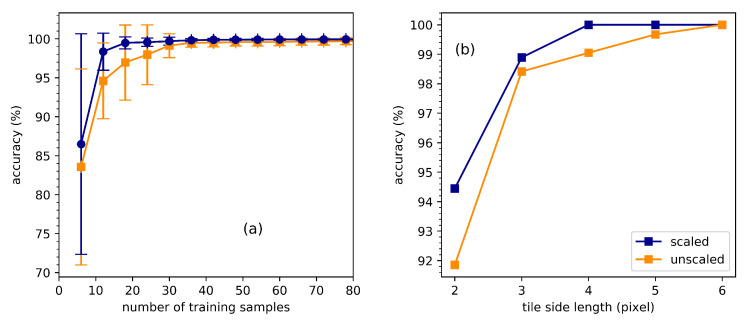
Dependence of the accuracy of the classification on: the number of samples in the training set (**a**); and the tile side length (**b**). The blue line denotes the accuracy with scaling, while the orange line denotes the accuracy without scaling. In (**a**), the tile side length is set to 4 and, in (**b**), the number of training samples is set to 216.

**Figure 4:**
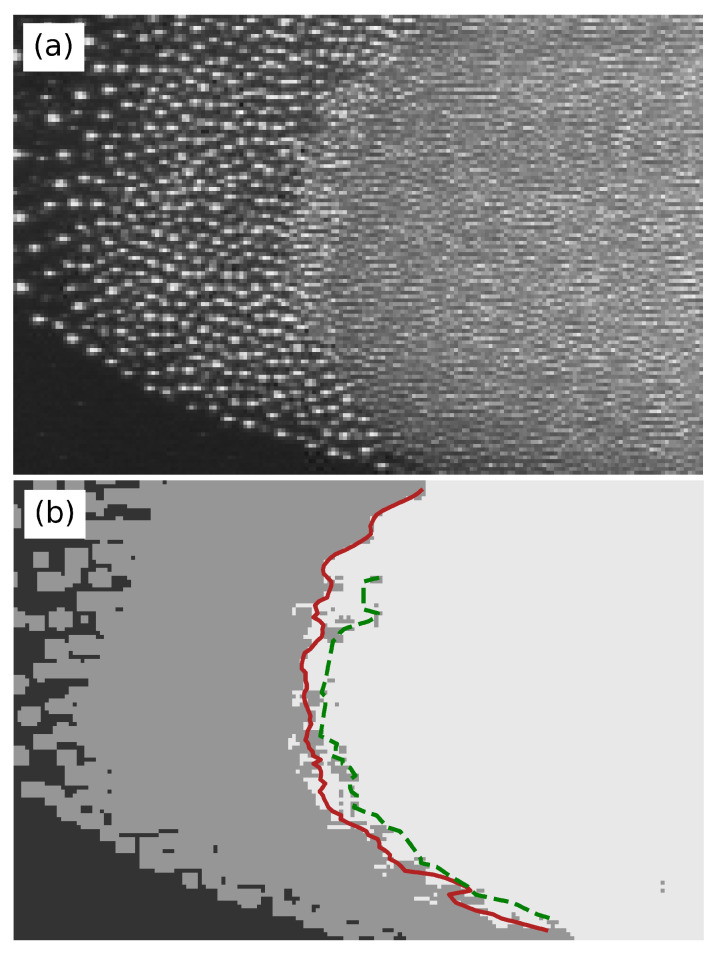
Original image selected from the experiment recording (**a**); and the results obtained by the SVC classifier (**b**). Area of small particles (white), big particles (gray), and the background microparticle-free plasma (black) are clearly distinguished. The red and green curves indicate the interface detected with two different methods (see text).

**Table 1:** A few samples of the scaled training set.

No.	Variance	Mean	Label
1	0.184	0.893	1
2	0.000	0.019	3
3	0.201	0.915	1
4	0.998	0.712	2
5	0.702	0.477	2
⋮	⋮	⋮	⋮
